# Autonomic nervous system and lipid metabolism: findings in anxious-depressive spectrum and eating disorders

**DOI:** 10.1186/1476-511X-10-192

**Published:** 2011-10-28

**Authors:** Elisabetta Pistorio, Maria Luca, Antonina Luca, Vincenzo Messina, Carmela Calandra

**Affiliations:** 1 Department of Medical and Surgery Specialties-Psychiatry Unit, University Hospital "Policlinico-Vittorio Emanuele", (Via S. Sofia 78), Catania, (95100), Italy; 2 Department of Neuroscience, University Hospital "Policlinico-Vittorio Emanuele", (Via S. Sofia 78), Catania, (95100), Italy

**Keywords:** depression, anxiety, anxious-depressive disorder, eating disorders, lipid metabolism, autonomic nervous system functioning, lipid index

## Abstract

**Objective:**

To correlate lipid metabolism and autonomic dysfunction with anxious-depressive spectrum and eating disorders. To propose the *lipid index *(LI) as a new possible biomarker.

**Methods:**

95 patients and 60 controls were enrolled from the University Psychiatry Unit of Catania and from general practitioners (GPs). The patients were divided into four pathological groups: Anxiety, Depression, Anxious-Depressive Disorder and Eating Disorders [Diagnostic and Statistical Manual of Mental Disorders Fourth Edition Text Revision (DSM-IV-TR) official/appendix criteria]. The levels of the cholesterol, triglycerides and apolipoproteins A and B were determined. The LI, for each subject, was obtained through a mathematical operation on the values of the cholesterol and triglycerides levels compared with the maximum cut-off of the general population. The autonomic functioning was tested with Ewing battery tests. Particularly, the correlation between heart rate variability (HRV) and lipid metabolism has been investigated.

**Results:**

Pathological and control groups, compared among each other, presented some peculiarities in the lipid metabolism and the autonomic dysfunction scores. In addition, a statistically significant correlation has been found between HRV and lipid metabolism.

**Conclusions:**

Lipid metabolism and autonomic functioning seem to be related to the discussed psychiatric disorders. LI, in addition, could represent a new possible biomarker to be considered.

## Background

The dysfunctions of lipid metabolism and autonomic nervous system have been found to be linked with anxious-depressive spectrum and eating disorders [1-19], but no definitive conclusions has yet been reached.

The aim of this work is to correlate the levels of cholesterol, triglycerides, apolipoproteins A-B, LI and autonomic dysfunction to depression, anxiety, anxious-depressive disorder and eating disorders. In addition, the correlation between HRV and lipid metabolism has been tested.

## Methods

95 patients (32 men and 63 women, mean age 46.0 SD ± 15.5) and 60 controls (36 men and 24 women, mean age 50.5 SD ± 10.8) were enrolled from the University Psychiatry Unit of Catania and from GPs. The demographic characteristics of the pathological and control groups are reported in Table 1.

**Table 1:** Demographic characteristics of the sample

Group	N	%	Age
**Depression**	39		50 ± 14.8

M	9	23%	49.8 ± 14.3

F	30	77%	50 ± 14.0


**Anxiety**	28		48.0 ± 17.7

M	16	57%	48.6 ± 16.2

F	12	43%	47.1 ± 20.2


**Anxious-depressive disorder**	13		46.6 ± 13.8

M	6	54%	52.8 ± 10.6

F	7	46%	41.4 ± 14.7


**Eating Disorders**	15		31.3 ± 7.06

M	1	7%	24

F	14	93%	31.8 ± 7.02


**Controls**	60		50.5 ± 10.8

M	36	60%	50.6 ± 9.9

F	24	40%	50.3 ± 12.3

**Legend: **The table shows data regarding the male (M) and female (F) distribution within every group, in number (N) and percentage (%). In addition, age is reported, referring to the whole group and to the gender [mean age and standard deviation (±)].

The local ethics committee approved this study (n. 321 register of trials), and all participants signed an informed consent.

Subjects with familial dyslipidemia, heart disease and psychotic disorders were excluded from the study.

Four pathological groups were created: Anxiety, Depression, Anxious-Depressive Disorder and Eating Disorders (ED) (Anorexia, Bulimia, Binge Eating Disorder). The diagnoses were formulated according to the criteria of the DSM-IV-TR (official/appendix criteria)[20].

**Figure 1:**
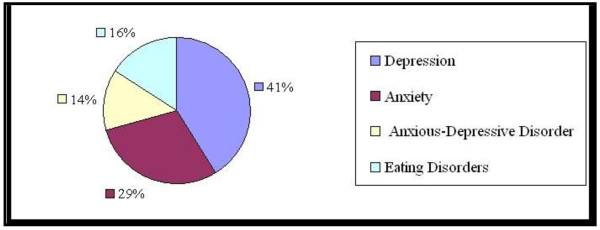
**(Distribution of pathological groups)-Legend: **the percentages of the pathological groups within the sample are shown.

Figure 1 shows the distribution of the pathological groups.

The autonomic nervous system (ANS) functioning was evaluated with the Ewing battery of tests through an electrocardiograph and a sphygmomanometer; particularly: Deep Breathing, Lying-to-Standing, Valsalva Manoeuvre and Sustained Handgrip tests [21,22]. Scores from 1 (mild) to 5 (severe) were considered parameters of autonomic dysfunction, passing through intermediate values of severity [23].

Particularly, the correlation between HRV (time-domain method) and lipid metabolism has been investigated.

Cholesterol, triglycerides and apolipoproteins A and B were analyzed through fasting blood samples. All the analyses were repeated three times.

Normal fasting rates of triglycerides range from 50 to 160 mg/dl; normal fasting rates of cholesterol range from 100 to 200 mg/dl [24].

All the subjects' rates of cholesterol and triglycerides were compared to the maximum cut-off of the general population (subject cholesterolemia/200; subject trigliceridemia/160) thus creating two ratios: cholesterol index (CI) and triglycerides index (TI).

Through the operation CI - TI the LI was obtained. A positive index number means that the cholesterol production is prevalent, while a negative one indicates a prevalent triglycerides production.

A value of + 0,15 or - 0,15 could be taken as 0, since it could be considered an irrelevant nutritional variation.

### Data analysis

The Statistical Package for Social Sciences (SPSS, 2000) was used. T-test and Z test were used. The association between categorical descriptive variables was tested through paired-samples t-test.

Mann-Whitney test was used to compare the results regarding the Ewing score. Pearson correlation has been performed to find possible correlations between HRV and lipid metabolism. The p values were then obtained with t-test. Values of p ≤0.05, < 0.01 and < 0.001 were considered statistically significant.

## Results

### Lipid Metabolism

#### Cholesterol

The anxious-depressive disorder group, compared to all the other groups (anxiety: t = 2,27, p < 0,025; depression: t = 3,42, p < 0,005; ED: t = 2,94, p < 0,005), and to the control group(t = 1,75, p < 0,05), presented lower levels of cholesterol.

With regards to the other groups, their cholesterol levels were higher compared to the control group, but there was not a statistically significant difference between the pathological groups compared among each other.

#### Triglycerides

The anxiety and anxious-depressive disorder groups, compared among each other, did not present a statistically significant difference in the triglycerides levels, but they both presented significantly higher triglycerides levels compared to all the other groups and to the control group (the anxiety group versus: depression: t = 4,5, p < 0,005; ED: t = 6,31, p < 0,05; control group: t = 5,41, p < 0,005); (the anxious-depressive disorder group versus: depression: t = 6,7, p < 0,005; ED t = 10,7, p < 0,005; control group: t = 2,91, p < 0,005).

The depression group presented lower triglycerides levels, when compared to anxiety, anxious-depressive disorder and control groups (Z = 4,46, p < 0,001); higher triglycerides levels, when compared to the eating disorders group (t = 3,36, p < 0,005), which presented lower triglycerides levels than in all the pathological and control groups (t = 8,31, p < 0,005).

#### LI

The eating disorders group presented the highest LI among the pathological and control groups (depression: t = 4,75, p < 0,005; anxiety: t = 5,87, p < 0,005; anxious-depressive disorders: t = 14, p < 0,005; control group: t = 12,5, p < 0,005). The LI, among the anxiety and anxious-depressive disorder groups, did not present a statistically significant difference, but it was significantly lower than that of the control group and of the other pathological groups (t = 2, p < 0,05). In terms of average, the anxious-depressive disorder group presented the lowest LI of the whole sample of the study.

The depression group presented, therefore, a higher LI than did the anxiety (t = 3.5, p < 0.005) and anxious-depressive disorder groups (t = 9,25, p < 0,005), but did not present a statistically significant difference compared to the control group.

#### Apolipoproteins A and B

The general population maximum cut-off was not exceeded either in the pathological groups or in the control one. Nevertheless, the anxious-depressive disorder group presented higher levels of Apolipoproteins A, compared to the other groups and to the control group. This same pattern was observed for Apolipoproteins B, only in the anxiety group.

For more details about the results regarding lipid metabolism, see Table 2.

**Table 2:** Demographic data and serum lipid levels in pathological and control groups

Group	Age	TC	TG	LI	ApoA	ApoB
**Depression**	50 ± 14.8	202.4 ± 49.6	88.1 ± 36.2	0.46 ± 0.2	155.7 ± 39.1	125.1 ± 49.7

M	49.8 ± 14.3	171.2 ± 40.5	93.7 ± 34.0	0.27 ± 0.1	161.3 ± 36.6	98.7 ± 11.9

F	50 ± 14.0	211.0 ± 48.8	86.4 ± 37.3	0.52 ± 0.2	154.1 ± 40.3	133.0 ± 54.0


**Anxiety**	48.0 ± 17.7	190.7 ± 47.2	181.2 ± 81.5	-0.18 ± 0.4	161.9 ± 56.9	169.9 ± 32.9

M	48.6 ± 16.2	201.0 ± 34.2	195.3 ± 93.3	-0.22 ± 0.5	154.5 ± 56.4	172.8 ± 28.8

F	47.1 ± 20.2	177 ± 59.3	162.5 ± 61.3	-0.13 ± 0.24	167.5 ± 59.2	166.1 ± 38.6


**Anxious-depressive disorder**	46.6 ± 13.8	160.9 ± 28.1	143.7 ± 19.5	-0.09 ± 0.10	189.3 ± 41.1	125.9 ± 42.2

M	52.8 ± 10.6	148.3 ± 15.6	134 22.8	-0.09 ± 0.12	170.8 ± 54.8	114.1 ± 43.1

F	41.4 ± 14.7	171.7 ± 33.0	152.1 ± 12.3	-0.10 ± 0.08	205.1 ± 15.9	136 ± 41.9


**Eating Disorders**	31.3 ± 7.06	197 ± 31.48	53.5 ± 25.6	0.65 ± 0.1	130.9 ± 44.5	156.5 ± 35.8

M	24	201	49	0.07	106	170

F	31.8 ± 7.02	197 ± 32.65	53.8 ± 26.6	0.65 ± 0.1	132.7 ± 45.7	155.5 ± 36.9


**Controls**	50.5 ± 10.8	177.9 ± 19.9	119 ± 27.9	0.15 ± 0.2	135.7 ± 42.2	145.8 ± 33.5

M	50.6 ± 9.9	177.6 ± 18.3	123.6 ± 29.2	0.12 ± 0.2	146.4 ± 44.9	140.8 ± 36.0

F	50.3 ± 12.3	178.0 ± 12.3	112 ± 24.8	0.19 ± 0.1	119.7 ± 32.4	153.4 ± 28.5

**Legend: **Data regarding age and lipid blood levels of every group are reported [as mean and standard deviation(±)], referring to the entire group and to the gender. M = male; F = female; TC = total cholesterol; TG = triglycerides; LI = lipid index; ApoA and ApoB = apolipoproteins A and B.

### Autonomic Nervous System Functioning

**Figure 2:**
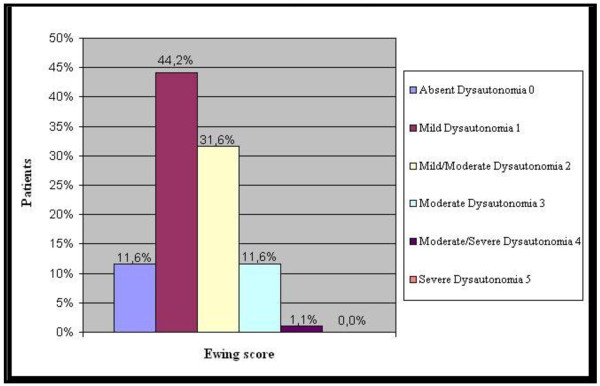
**(Ewing score of patients)-Legend: **the distribution of the different levels of the autonomic dysfunctions within the whole pathological sample is shown in percentage.

Among the pathological groups, the most represented autonomic dysfunctions were at a mild-mild/moderate level (see Figure 2). Among the control group, 51.7% did not present autonomic dysfunctions, 33.3% presented mild dysautonomia and 15.0% presented mild/moderate dysautonomia. In contrast to the pathological groups, the control group did not present other, more severe, dysautonomia levels. All the pathological groups presented a statistically significant difference compared to the control group considering mild/moderate dysautonomia (Mann Whitney test for n > 20, Z = 30 p < 0,001). In terms of average, the highest Ewing score was in the anxious-depressive disorder group. For more details, see Table 3.

**Table 3:** Sample's Ewing score

Group	Ewing score
**Depression**	1.3 ± 0.8

M	1.4 ± 1.3

F	1.3 ± 0.7


**Anxiety**	1.3 ± 0.9

M	1.1 ± 0.8

F	1.5 ± 0.9


**Anxious-depressive disorder**	2 ± 0.9

M	2.1 ± 0.9

F	1.8 ± 0.8


**Eating Disorders**	1.5 ± 0.6

M	2

F	1.5 ± 0.6


**Controls**	0.6 ± 0.7

M	0.7 ± 0.7

F	0.4 ± 0.5

**Legend: **The Ewing score of each group is reported [mean and standard deviation(±)], referring to the entire group and to the gender. M = male; F = female.

With regards, in particular, to HRV, there was not a statistically significant difference among the pathological groups (p > 0,1). When compared to the control group, only the depression and anxiety groups presented a statistically significant difference (p = 0,05). The anxious-depressive group, in terms of mean value, is the group with the lowest HRV of the whole sample (33,1 ± 10,2). Considering the whole sample, the correlation between ANS and lipid metabolism has been investigated, referring in particular to HRV, with highly statistically significant results (p < 0,01). HRV and LI presented a positive correlation: the anxious-depressive disorder group, in fact, presented the lowest LI (showing to be the group with the most severe triglycerides hyperproduction) and the lowest HRV. Correspondingly, this group was the one with the highest average of Ewing score. HRV has been found to be inversely related to the triglycerides levels and positively related to the cholesterol levels; these results confirmed the HRV-LI correlation. Apolipoproteins A presented a negative correlation to HRV. The opposite has been found for Apolipoproteins B. For more details, see Table 4.

**Table 4:** HRV and Lipid metabolism

Group	HRV	correl.HRV-LI	correl.HRV-TC	correl.HRV-TG	correl.HRV-ApoA	correl. HRV-ApoB
**Depression**	36.8 ± 11.1					
					
					
**Anxiety**	35.6 ± 10.7					
					
					
**Anxious-depressive disorder**	33.1 ± 10.2	0.67 p < 0,01	0.75 p < 0,01	-0.57 p < 0,01	-0.94 p < 0,01	0.32 p < 0,01
					
					
**Eating Disorders**	37.1 ± 13.7					
					
					
**Controls**	37.2 ± 15					

**Legend: **Data regarding Heart rate variability (standard deviation of all normal RR intervals) of every group are reported [mean and standard deviation(±)]. In addition, the values of the correlation (correl.HRV-...) between Heart rate variability and the other variables reported are shown (referring to the whole sample), with the p values. HRV = Heart rate variability; LI = Lipid index; TC = Total cholesterol; TG = Triglycerides; ApoA and ApoB = Apolipoproteins A and B.

## Conclusions

Lipid metabolism and autonomic functioning seem to be related to the discussed psychiatric disorders.

In a context of enhancing knowledge, the lipid index could represent another biomarker to be taken into consideration.

Our findings are far from being definitive, also considering the small sample size and some disparities in male and female distribution. Further studies are necessary.

## Abbreviations

LI: Lipid index; GPs: general practitioners; DSM- IV-TR: Diagnostic and Statistical Manual of Mental Disorders, Fourth Edition, Text Revision; HRV: Heart rate variability; ED: Eating Disorders; ANS: Autonomic nervous system; CI: Cholesterol index; TI: Triglycerides index.

## Competing interests

The authors declare that they have no competing interests.

## Authors' contributions

EP collected data, interpreted the results, and helped draft the manuscript.

ML, AL, VM assisted with collecting data, interpreting the results and drafting the manuscript. CC designed the study, interpreted the results, and helped draft the manuscript. All authors read and approved the final manuscript.
